# Stiff, light, strong and ductile: nano-structured High Modulus Steel

**DOI:** 10.1038/s41598-017-02861-3

**Published:** 2017-06-05

**Authors:** H. Springer, C. Baron, A. Szczepaniak, V. Uhlenwinkel, D. Raabe

**Affiliations:** 1 Max-Planck-Institut für Eisenforschung GmbH, 40237 Düsseldorf, Germany; 2 Foundation Institute of Material Science (IWT), Department of Production Engineering, University of Bremen, Badgasteiner Straße 3, 28359 Bremen, Germany

## Abstract

Structural material development for lightweight applications aims at improving the key parameters strength, stiffness and ductility at low density, but these properties are typically mutually exclusive. Here we present how we overcome this trade-off with a new class of nano-structured steel – TiB_2_ composites synthesised *in-situ* via bulk metallurgical spray-forming. Owing to the nano-sized dispersion of the TiB_2_ particles of extreme stiffness and low density – obtained by the *in-situ* formation with rapid solidification kinetics – the new material has the mechanical performance of advanced high strength steels, and a 25% higher stiffness/density ratio than any of the currently used high strength steels, aluminium, magnesium and titanium alloys. This renders this High Modulus Steel the first density-reduced, high stiffness, high strength and yet ductile material which can be produced on an industrial scale. Also ideally suited for 3D printing technology, this material addresses all key requirements for high performance and cost effective lightweight design.

## Introduction

Lightweight design is the major frontier to increase performance and efficiency of transportation systems and machines. The main selection criterion for load bearing materials is their strength, which allows for reducing the wall thickness and thus weight of the components. As nearly all components of machines and transportation systems are intended to bear load without permanent plastic deformation, their yield strength (YS) is more important than the ultimate strength (UTS). Ductility, i.e. the ability to undergo plastic deformation (here noted as the total elongation; TE), is vital for forming operations during the manufacturing of parts and as a safety reserve for accidental overloading for example in a crash situation^1^.

**Figure 1:**
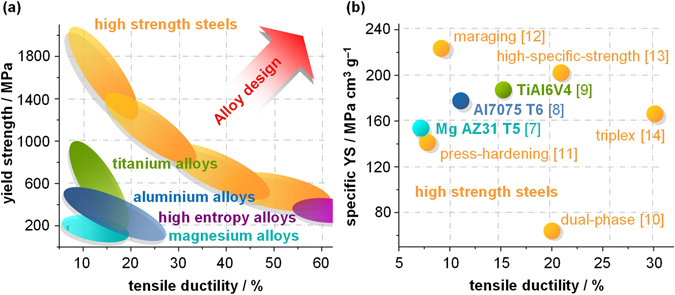
Established Ashby-type diagrams for lightweight material design: (**a**) Areas of YS vs. TE for different material systems^2–6^. (**b**) YS per ρ vs. TE of the highest performing alloys of each material system^7–14^.

Figure 1a shows that advanced iron (Fe) based alloys, i.e. high strength steels, with their fine-tuned micro- and nano-structures offer a unique combination of strength and ductility often superior to aluminium (Al), magnesium (Mg) or titanium (Ti) systems and even to new material concepts such as high entropy alloys^2–6^. However, the elevated strength of steels comes at the price of high density (ρ). Consequently the specific YS (i.e. YS/ρ) of high performance Mg^7^, Al^8^ and Ti alloys^9^ is often the more adequate measure for comparing them to high strength steels commonly used in the automotive industry, such as dual-phase^10^ or press-hardening steels^11^ (Fig. 1b). Only highly alloyed and thus expensive steel concepts such as maraging^12^ or the recently developed lightweight steels^13, 14^, which are based on significant density-reduction via additions of up to 12 wt.% Al, show corresponding superior property combinations when related to ρ.

Focussing exclusively on the further improvement of YS, ρ and TE, though, neglects another important design key factor, namely the stiffness of materials, expressed by their Young’s modulus (*E*). It determines the deformation of a given part in the elastic regime irrespective of its strength, and thus for example the buckling resistance of an aeroplane wing or the deflection of a transmission part^1^. However, this most critical material property is often simply taken as a given intrinsic property, which can only be affected marginally – and often detrimentally – by alloying additions and mechanical or thermal processing. In Fe and Al alloys, for example, only chromium and rhenium or lithium, respectively, are known to raise the stiffness slightly, while almost all other alloying elements decrease it^15, 16^. The presence of strengthening defects such as dislocations and internal interfaces also typically lowers the stiffness^17^. Interestingly, the specific modulus, i.e. the *E*/ρ ratio, of all established metallic structural materials – from Mg alloys to lightweight steels – is with about 26 GPa cm^3^ g^−1^ almost identical. As low *E* values need to be compensated by a larger wall thickness, the original weight saving potential provided by established “light” materials is thereby often eliminated.

A promising pathway to overcome these alloy and design limits, and in particular to raise the specific modulus, lies in creating composite microstructures, i.e. blending light and stiff particles into strong and ductile metallic matrices^18^. Corresponding Fe-based composites, also termed High Modulus Steels (HMS), are especially attractive because of the possibility to exploit the numerous phase transformations of steels to tune the mechanical performance, and keep the raw material and production costs to a minimum^19, 20^. Ti-diboride (TiB_2_) is an ideally suited particle phase, as it is not only very effective in the current context (*E* of 515 GPa and ρ of 4.52 g cm^−3^)^21^ but also enables synthesis of such composites *in-situ* via liquid metallurgy^22, 23^, a key factor for cost efficient mass production. However, while Fe – TiB_2_ based composites indeed allow achieving favourable physical properties, their mechanical performance is yet unsatisfactory, i.e. both strength and ductility have so far been too low compared to other lightweight materials^24^. This is due to the formation of comparably large (several µm^2^) and inherently brittle particles of detrimental morphology, promoting crack initiation^25^. Those particles precipitate from the homogeneous liquid phase as thermodynamically stable solidification products, and therefore cannot be refined via established dissolution and precipitation procedures, as applied for example to carbides in tool steels or intermetallic phases in Al alloys^19^.

### Objective

We have developed a new combined alloy and synthesis concept which makes it possible to overcome this trade-off between excellent physical properties and detrimental mechanical performance. Our approach is based on coupling the *in-situ* formation of TiB_2_ of suited alloy compositions with efficient microstructure refinement through accelerated solidification kinetics, in a setup which is capable of mass production. The resultant nano-structured Fe – TiB_2_ HMS is of dramatically improved strength compared to conventional steel composites without sacrificing ductility, while exhibiting a higher stiffness/density ratio than any of the currently used high performance materials.

### Experimental details

#### Synthesis and processing

Our concept is demonstrated on an Fe – 6.38 Ti – 2.4 B alloy (wt.%), which corresponds to about 13 vol.% of TiB_2_ in a ferritic Fe matrix. While larger particle fractions can be easily achieved through higher Ti and B concentrations, the chosen eutectic composition exhibits with ~1400 °C the lowest melting temperature in the Fe – TiB_2_ pseudo-binary system^26^, and thus more cost effective production. A charge of 4 kg based on pure metals were molten in a vacuum induction furnace under argon (Ar) atmosphere and cast into a cylindrical copper moulds of 30 mm internal diameter, with a cooling rate of about 10 K s^−1^, which is comparable to conventional steel production conditions (solidification rate of about 5 to 10 K s^−1^) such as in continuous casting^27^.

**Figure 2:**
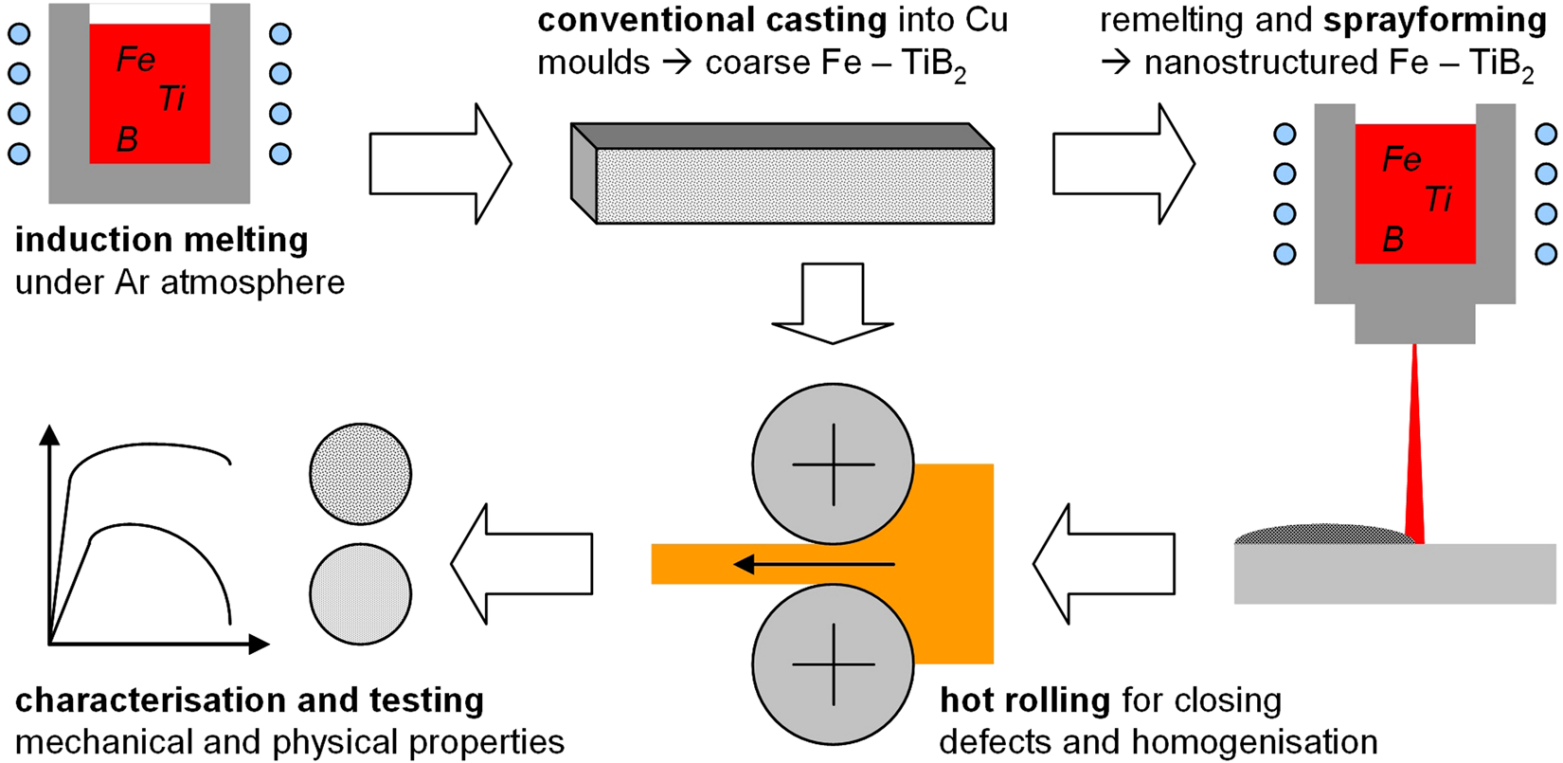
Sketch of the synthesis and processing routes used for the High Modulus Steels presented in this study.

We identified spray-forming as the optimum synthesis technique to achieve the desired massive microstructure refinement of the composite material, as it represents an industrially established liquid metallurgy synthesis technique capable of high volume production at very fast solidification kinetics. A 200 mm long section (about 1.15 kg) was re-molten under Ar (melt temperature about 1550 °C, i.e. 150 °C above the liquidus temperature) and sprayed with 20 bar Ar pressure and a deposition rate of about 72 kg h^−1^ onto a rotating tube (low C steel, 90 mm outer diameter, sandblasted surface, positioned 140 mm below the spray nozzle). The spray-forming process exhibits three differing cooling rates, i.e. during flight of the droplets, during impact and on the substrate. In the upper temperature regime, relevant for the solidification and thus for the formation and growth of the TiB_2_ particles from the liquid during the first two solidification phases, the cooling rate is in the order of 10^4^ K s^−1^ ^28^. Subsequent to the deposition the tube-substrate was removed by lathing, leaving a ring of about 20 mm width and 8 mm thickness. An 80 mm long section of the spray-deposited ring was hot rolled together with a section of the as-cast billet (machined to identical cross-section as the sprayed ring) to a thickness of 2 mm at 1100 °C and cooled at air to room temperature. Hot rolling was performed to remove any porosity and non-equilibrium metastable phases possibly left after the primary synthesis. Both conventional and spray-formed materials are thus of identical chemical composition, but underwent different solidification kinetics. A sketch of the synthesis and processing chain is shown in Fig. 2.

#### Characterisation

Microstructures of both materials were characterised on cross sections prepared by standard metallographic techniques with scanning electron microscopy (SEM; Zeiss Merlin with a Gemini-type field emission gun). Phase identification for conventional HMS was performed with electron backscatter diffraction analysis (EBSD; EDAX detector and TSL OIM 7.2 software) in the SEM. The nano-structured HMS was characterised using transmission electron microscopy (TEM; Jeol JEM 2200FS operated at 200 kV on samples prepared with a focussed ion beam system (FIB; FEI Helios Nanolab 600i). *E* values were determined by a GrindoSonic MK5 “Industrial” excitation system (flexural vibration resonance) on rectangular specimens (20 mm long, 5 mm wide), while ρ values were measured on machined chippings under protective atmosphere in a gas pycnometer (Micromeritics Accupyc 1330). Tensile testing was performed parallel to the rolling direction on dog-bone shaped samples with gauge dimensions of 5 mm length and 2 mm width at an initial strain rate of 10^−3^ s^−1^ with digital image correlation to assess the strain.

## Results

**Figure 3:**
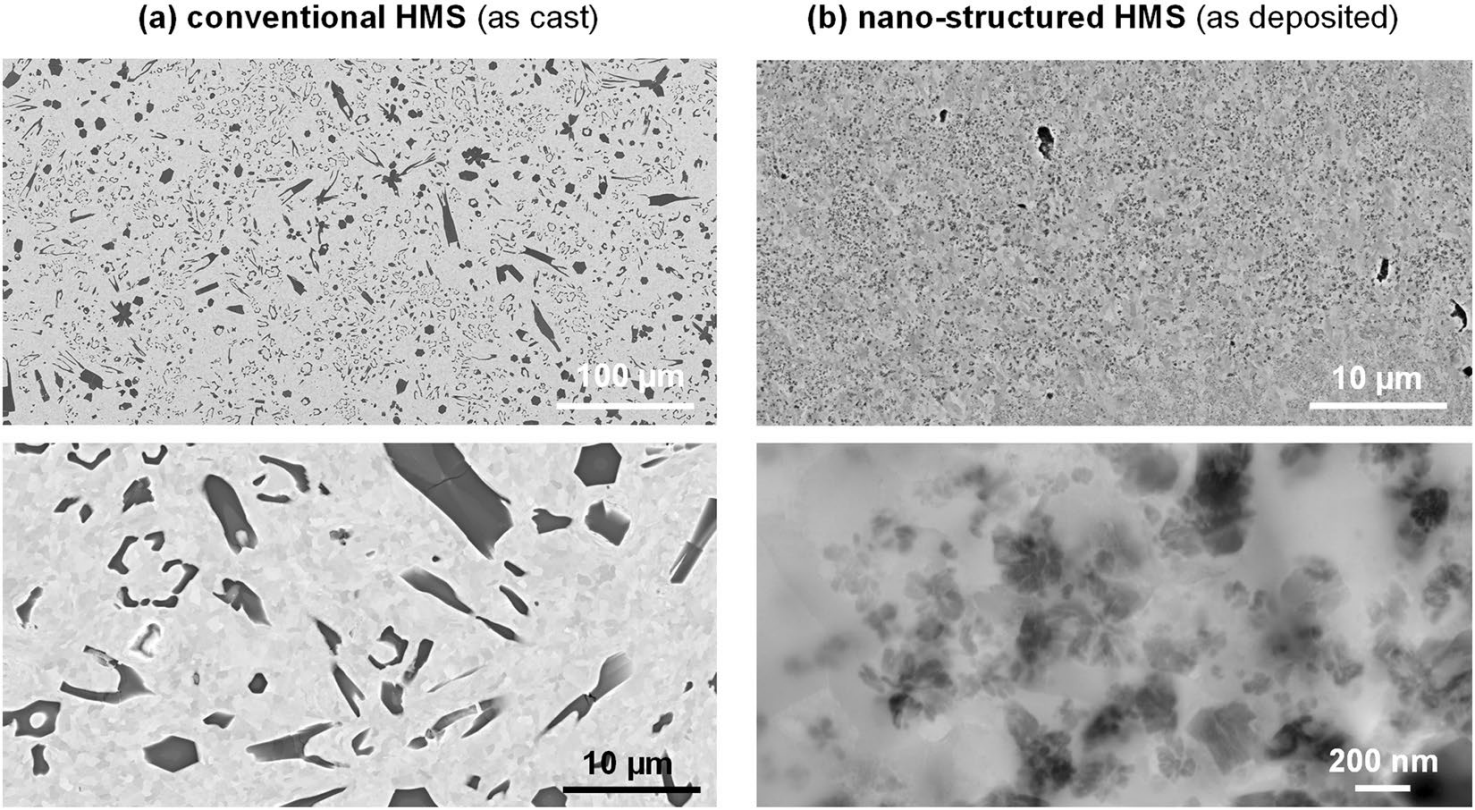
Microstructure characterisation results as SEM BSE and STEM BF images of High Modulus Steels after the primary synthesis by (**a**) conventional casting and (**b**) spray-forming.

After the primary synthesis (Fig. 3) the conventionally produced HMS (Fig. 3a) exhibits a typical as-cast microstructure^25^ with about 15 vol.% of TiB_2_ particles of irregular shape and size (1–15 µm in diameter) and number density of about 0.06 µm^−2^ evenly dispersed in a ferritic matrix with a typical widely irregular α-Fe grain size distribution. The spray-formed material (Fig. 3b) exhibits microstructural features on a nano-metric scale. Only few pores can be observed, and the fine grained ferritic matrix contains a high number of darker features of irregular morphology, corresponding to TiB_2_ particles as well as metastable phases.

**Figure 4:**
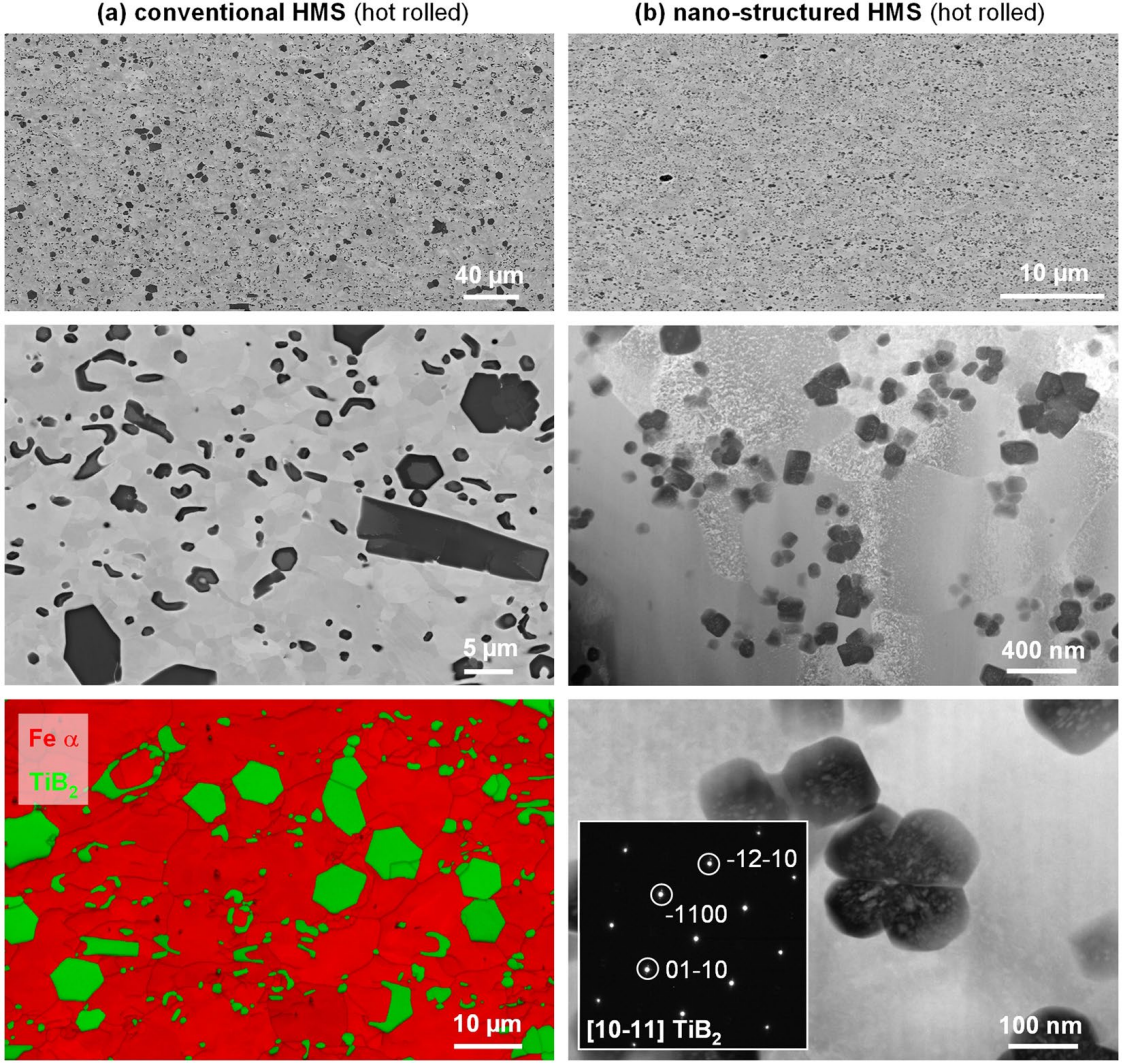
Microstructure characterisation results as SEM BSE / EBSD and STEM DF images of High Modulus Steels after hot rolling for (**a**) conventional casting and (**b**) spray-forming. The effective nano-structuring of both particles and matrix through spray-forming is evident.

After hot rolling (Fig. 4), the microstructure of the conventionally produced HMS remains virtually unchanged, with only slight spheroidisation of the TiB_2_ particles and recrystallization of the ferritic matrix to a grain size of about 15 µm (Fig. 4a). The rapidly solidified spray-formed material, on the other hand, remains nano-structured (Fig. 4b) with a ferritic matrix grain size of about 400 nm. Furthermore, no metastable phase could be detected anymore, but a high number density (12.87 µm^−2^) of extremely small (about 50–200 nm in diameter) and spherical TiB_2_ particles of similar volume fraction as the conventional material. High resolution characterisation reveals that some of the TiB_2_ particles appear as small clusters, possibly as an effect of sympathetic nucleation, and contain Fe-rich inclusions.

**Figure 5:**
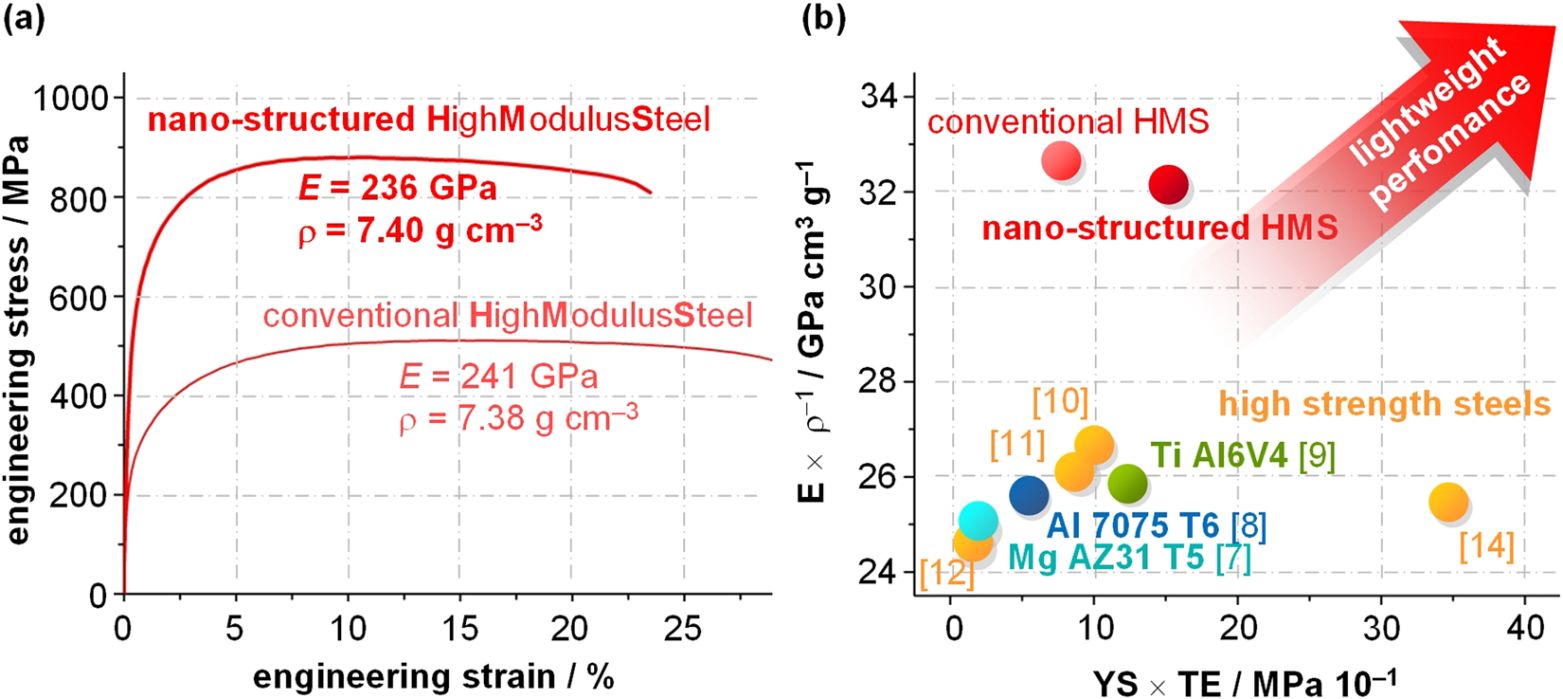
(**a**) Tensile testing results and physical properties of conventional and novel nano-structured High Modulus Steels. (**b**) Materials property map of all key factors for lightweight design showing the unique property profile of nano-structured High Modulus Steels.

The very efficient nano-structuring achieved by the rapid solidification in the spray-formed material translates into an extremely favourable property profile (Fig. 5a). Compared to the conventionally produced reference material, both YS and UTS are about doubled without sacrificing TE, reaching the mechanical property level of advanced high strength dual-phase steels (Fig. 1a), yet at 5% reduced ρ and 13% higher *E*. The pronounced strengthening is not only caused by the refined particles, which are now small enough to effectively interact with dislocations as the carriers of plastic deformation, but also through the severe reduction of the matrix grain size (Hall-Petch effect). The latter mechanism is especially important as it has the advantage of improving the materials strength without lowering its impact toughness^19^. Strengthening of conventional Fe – TiB_2_ composites through additional plastic deformation such as cold rolling is difficult, as the larger particles easily fracture in the process and thus the material embrittles.

## Discussion

Despite the comparatively lean alloy composition and simple synthesis suitable for mass production, the property profile of the here presented nano-structured HMS is superbly suited to open novel pathways for lightweight design. The observed favourable mechanical properties appear to be mainly caused by the pronounced size reduction and spheroidisation of the *in-situ* formed TiB_2_ particles through the rapid solidification kinetics of spray-forming. While the volume fraction of the particles remains similar, there is now much less interfacial stress concentration compared to the conventionally produced alloys with their much larger and sharp-edged particles (which are also often clustered and pre-cracked). Furthermore, the number density of particles is substantially increased, as is the density of grain boundaries within the also refined matrix, and the particles are small enough to effectively interact with dislocations during deformation. All those factors together appear to enable to increase strength without deteriorating ductility of the composite structure. The effective contribution of each individual mechanism to this property improvement, though, requires more in-depth investigations in future works with *in-situ* microscopy and concerted modelling techniques, in order to fully clarify the underlying microstructural phenomena.

Figure 5b summarises all material properties which are essential for evaluating a material’s suitability for weight reduction^1^. We have chosen tensile ductility (i.e. the total elongation in tensile testing) as it comprises the full deformation regime and thus also gives an insight into the damage tolerance of materials. The map reveals that all other high performance materials cover a wide range of mechanical properties, yet have a very similar stiffness/density ratio. The nano-structured High Modulus Steel, on the other hand, has not only excellent mechanical properties, but its about 25% increased specific modulus offers up to now entirely untapped potential for realising weight-critical applications: The energy efficiency of a wind turbine for example can be increased as the stiffer material offers a higher resistance against buckling and thus the tower can be build higher. The drive shaft and rotors located at the top can be lightened at identical elastic and plastic deflections, saving material and effort for installation as well as increasing their performance.

The property profile of nano-structured HMS can be even further improved in the future, as alloying additions can be utilised to increase its strength (e.g. via small amounts of other precipitating phases, albeit at reduced ductility^29^) and enable functional properties such as increased corrosion resistance (e.g. Cr and Mo additions). Interestingly, these materials are also ideally suited for the rapidly growing field of additive manufacturing of near-net shaped parts, as the high cooling rates of the commonly used Laser-based 3D-printing devices should enable to achieve similar microstructures as in our spray-forming process^30, 31^. While certainly less cost efficient than large volume liquid metallurgy processes such as block- or strip-casting, spray-forming is typically of less effort than solid state powder metallurgy processes utilised for composite material synthesis. The alloying costs associated with the comparatively large Ti concentrations can be significantly reduced by utilising *in-situ* reduction of Ti oxides in the melt^32^. Nano-structured HMS thus open a wide spectrum of novel pathways towards the next generation of materials for lightweight design: stiff and of low density, but strong and ductile, while lean and cost effective.
